# 3,9-Di-2-furyl-2,4,8,10-tetra­oxa­spiro­[5.5]undecane

**DOI:** 10.1107/S1600536808032996

**Published:** 2008-10-18

**Authors:** Jun Lin, Fang-Fang Jian

**Affiliations:** a Microscale Science Institute, Department of Chemistry and Chemical Engineering, Weifang University, Weifang 261061, People’s Republic of China; b Microscale Science Institute, Weifang University, Weifang 261061, People’s Republic of China

## Abstract

The title compound, C_15_H_16_O_6_, was prepared by reaction of 2,2-bis­(hydroxy­meth­yl)propane-1,3-diol with 2-furaldehyde in the presence of hydro­chloric acid at room temperature. The asymmetric unit contains two crystallographically independent mol­ecules. In these two mol­ecules, the dihedral angles between the five-membered rings are 56.4 (3) and 56.3 (3)°. The six-membered rings adopt chair conformations. Inter­molecular C—H⋯π inter­actions link the mol­ecules and may be effective in the stabilization of the crystal structure.

## Related literature

For background on di-acetals of penta­erythritol, see: Jermy & Pandurangan (2005 ▶). For puckering parameters, see: Cremer & Pople (1975 ▶).
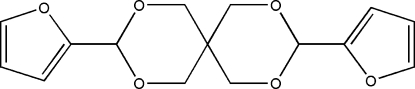


## Experimental

### 

#### Crystal data

C_15_H_16_O_6_
*M* *_r_* = 292.28
Orthorhombic, 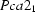

*a* = 11.756 (3) Å
*b* = 5.5832 (13) Å
*c* = 42.728 (9) Å
*V* = 2804.5 (11) Å^3^
*Z* = 8
Mo *K*α radiation
μ = 0.11 mm^−1^
*T* = 273 (2) K
0.20 × 0.15 × 0.13 mm

#### Data collection

Bruker SMART CCD area-detector diffractometer
Absorption correction: none
13819 measured reflections
3554 independent reflections
1793 reflections with *I* > 2σ(*I*)
*R*_int_ = 0.069

#### Refinement

*R*[*F* ^2^ > 2σ(*F* ^2^)] = 0.050
*wR*(*F* ^2^) = 0.125
*S* = 1.02
3554 reflections
380 parameters
1 restraint
H-atom parameters constrained
Δρ_max_ = 0.17 e Å^−3^
Δρ_min_ = −0.17 e Å^−3^

### 

Data collection: *SMART* (Bruker, 1997 ▶); cell refinement: *SAINT* (Bruker, 1997 ▶); data reduction: *SAINT*; program(s) used to solve structure: *SHELXS97* (Sheldrick, 2008 ▶); program(s) used to refine structure: *SHELXL97* (Sheldrick, 2008 ▶); molecular graphics: *SHELXTL* (Sheldrick, 2008 ▶); software used to prepare material for publication: *SHELXTL*.

## Supplementary Material

Crystal structure: contains datablocks global, I. DOI: 10.1107/S1600536808032996/at2637sup1.cif

Structure factors: contains datablocks I. DOI: 10.1107/S1600536808032996/at2637Isup2.hkl

Additional supplementary materials:  crystallographic information; 3D view; checkCIF report

## Figures and Tables

**Table 1:** Hydrogen-bond geometry (Å, °) *Cg*1 is the centroid of the O6*C*/C12*C*–C15*C* ring.

*D*—H⋯*A*	*D*—H	H⋯*A*	*D*⋯*A*	*D*—H⋯*A*
C1*A*—H1⋯*Cg*1	0.93	2.70	3.478 (6)	142

**Fig. 1.**
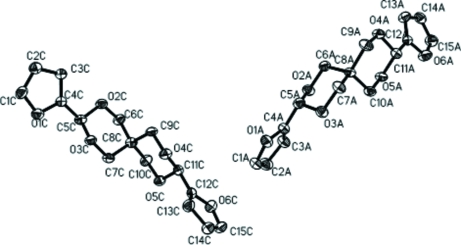
The molecular structure of (I) with the atom-labeling scheme. Displacement ellipsoids are drawn at the 30% probability level.

## supplementary crystallographic information

### Comment

The di-acetals of pentaerythritol are a series of useful organic compounds. They have been used as important intermediates in the synthesis of pesticides (Jermy & Pandurangan, 2005). we sythesis the title compound (I) and report its crystal structure here.

In the crystal structure of (I), the asymmetric unit contains two crystallographically independent molecules (Fig. 1). The dihedral angle formed by the ring (O1A/C1A–C4A) and the ring (O6A/C12A–C15A) is 56.4 (3)° and 56.3 (3)° for the ring (O1CA/C1C–C4C) and the ring (O6C/C12C–C15C). The six-membered rings of the two independent molecules of (I), (O2A/O3A/C5A–C8A), (O2C/O3C/C5C–C8C), (O4A/O5A/C8A–C11A) and (O4C/O5C/C8C–C11C) have chair conformations [the puckering parameters: Q_T_ = 0.564 (5) Å, φ = 170 (15)°, θ = 0.0 (5)°; Q_T_ = 0.563 (5) Å, φ = 244 (10)°, θ = 176.7 (5)°; Q_T_ = 0.574 (5) Å, φ = 292 (9)°, θ = 2.3 (5)° and Q_T_ = 0.573 (5) Å, φ = 309 (22)°, θ = 178.1 (5)°, respectively (Cremer & Pople, 1975)].

Intermolecular C···H···π link the molecules and may be effective in the stabilization of the crystal structure (Table 1).

### Experimental

The title compound (I) was prepared by the process as following: ethyl isonicotinate 1.51 g (0.01 mol) and hydrazine hydrate 0.32 g (0.01 mol) with ethanol at 377 K for 3 h, afford ivory-white compound A 1.32 g (yield 96%), then add 0.06 ml carbon disulfide and KOH 0.56 g(0.01 mol) with ethanol, stirred at room temperature for 5 h, afford yellow compound B 2.0 g (yield 85.6%). At last, add 0.32 g hydrazine hydrate to the compound B with water at 377 K for 12 h. Single crystals suitable for X-ray measurements were obtained by recrystallization from DMF-HCl(3:1) at 334 K.

### Refinement

H atoms were positioned geometrically and allowed to ride on their parent atoms, with C—H = 0.93–0.97 Å, and with *U*_iso_(H) = 1.2*U*_eq_ of the parent atoms. In the absence of significant anomalous scattering effects, Friedel pairs have been merged.

### Crystal data

**Table:**

C_15_H_16_O_6_	*F*(000) = 1232
*M**_r_* = 292.28	*D*_x_ = 1.384 Mg m^−^^3^
Orthorhombic, *P**c**a*2_1_	Mo *K*α radiation, λ = 0.71073 Å
Hall symbol: P 2c -2ac	Cell parameters from 2523 reflections
*a* = 11.756 (3) Å	θ = 1.9–28.8°
*b* = 5.5832 (13) Å	µ = 0.11 mm^−^^1^
*c* = 42.728 (9) Å	*T* = 273 K
*V* = 2804.5 (11) Å^3^	Bar, colourless
*Z* = 8	0.20 × 0.15 × 0.13 mm

### Data collection

**Table:**

Bruker SMART CCD area-detector diffractometer	1793 reflections with *I* > 2σ(*I*)
Radiation source: fine-focus sealed tube	*R*_int_ = 0.069
graphite	θ_max_ = 28.8°, θ_min_ = 1.9°
φ and ω scans	*h* = −15→15
13819 measured reflections	*k* = −7→6
3554 independent reflections	*l* = −28→57

### Refinement

**Table:**

Refinement on *F*^2^	Secondary atom site location: difference Fourier map
Least-squares matrix: full	Hydrogen site location: inferred from neighbouring sites
*R*[*F*^2^ > 2σ(*F*^2^)] = 0.050	H-atom parameters constrained
*wR*(*F*^2^) = 0.125	*w* = 1/[σ^2^(*F*_o_^2^) + (0.043*P*)^2^] where *P* = (*F*_o_^2^ + 2*F*_c_^2^)/3
*S* = 1.02	(Δ/σ)_max_ = 0.001
3554 reflections	Δρ_max_ = 0.17 e Å^−^^3^
380 parameters	Δρ_min_ = −0.17 e Å^−^^3^
1 restraint	Extinction correction: SHELXL97 (Sheldrick, 2008), Fc^*^=kFc[1+0.001xFc^2^λ^3^/sin(2θ)]^-1/4^
Primary atom site location: structure-invariant direct methods	Extinction coefficient: 0.0043 (7)

### Special details

**Table:**

Geometry. All e.s.d.'s (except the e.s.d. in the dihedral angle between two l.s. planes) are estimated using the full covariance matrix. The cell e.s.d.'s are taken into account individually in the estimation of e.s.d.'s in distances, angles and torsion angles; correlations between e.s.d.'s in cell parameters are only used when they are defined by crystal symmetry. An approximate (isotropic) treatment of cell e.s.d.'s is used for estimating e.s.d.'s involving l.s. planes.
Refinement. Refinement of *F*^2^ against ALL reflections. The weighted *R*-factor *wR* and goodness of fit *S* are based on *F*^2^, conventional *R*-factors *R* are based on *F*, with *F* set to zero for negative *F*^2^. The threshold expression of *F*^2^ > σ(*F*^2^) is used only for calculating *R*-factors(gt) *etc*. and is not relevant to the choice of reflections for refinement. *R*-factors based on *F*^2^ are statistically about twice as large as those based on *F*, and *R*- factors based on ALL data will be even larger.

### Fractional atomic coordinates and isotropic or equivalent isotropic displacement parameters (Å^2^)

**Table:**

	*x*	*y*	*z*	*U*_iso_*/*U*_eq_	
O1A	−0.9377 (3)	−0.0310 (7)	−0.19447 (12)	0.0640 (12)	
O2A	−0.7186 (2)	−0.2654 (6)	−0.14554 (7)	0.0511 (8)	
O3A	−0.8254 (3)	0.0757 (7)	−0.13372 (8)	0.0497 (8)	
O4A	−0.4986 (2)	−0.0918 (6)	−0.06685 (8)	0.0501 (8)	
O5A	−0.6606 (2)	−0.3243 (5)	−0.06050 (7)	0.0485 (8)	
O6A	−0.5577 (3)	−0.5078 (7)	−0.00488 (13)	0.0686 (14)	
C1A	−1.0251 (4)	−0.1649 (13)	−0.20675 (12)	0.0699 (17)	
H1	−1.0780	−0.1086	−0.2212	0.084*	
C2A	−1.0235 (5)	−0.3856 (14)	−0.19522 (15)	0.0729 (17)	
H2	−1.0737	−0.5093	−0.1999	0.087*	
C3A	−0.9290 (4)	−0.3953 (12)	−0.17413 (12)	0.0610 (14)	
H3A	−0.9060	−0.5269	−0.1624	0.073*	
C4A	−0.8807 (4)	−0.1810 (9)	−0.17450 (11)	0.0493 (13)	
C5A	−0.7824 (4)	−0.0747 (10)	−0.15763 (12)	0.0488 (12)	
H3	−0.7358	0.0191	−0.1722	0.059*	
C6A	−0.6229 (3)	−0.1810 (9)	−0.12730 (11)	0.0498 (12)	
H4	−0.5708	−0.0946	−0.1408	0.060*	
H5	−0.5825	−0.3170	−0.1186	0.060*	
C7A	−0.7335 (4)	0.1789 (9)	−0.11575 (12)	0.0529 (13)	
H6	−0.7644	0.2807	−0.0994	0.063*	
H7	−0.6864	0.2770	−0.1293	0.063*	
C8A	−0.6613 (4)	−0.0183 (8)	−0.10104 (15)	0.0406 (15)	
C9A	−0.5593 (4)	0.0911 (10)	−0.08414 (13)	0.0564 (13)	
H8	−0.5086	0.1644	−0.0993	0.068*	
H9	−0.5852	0.2148	−0.0699	0.068*	
C10A	−0.7312 (3)	−0.1575 (8)	−0.07684 (11)	0.0479 (12)	
H10A	−0.7649	−0.0464	−0.0620	0.058*	
H10B	−0.7923	−0.2426	−0.0873	0.058*	
C11A	−0.5709 (4)	−0.2040 (8)	−0.04513 (11)	0.0466 (11)	
H11A	−0.6017	−0.0853	−0.0305	0.056*	
C12A	−0.5016 (3)	−0.3830 (8)	−0.02793 (10)	0.0429 (11)	
C13A	−0.3940 (4)	−0.4551 (11)	−0.02931 (16)	0.0567 (16)	
H13A	−0.3380	−0.3968	−0.0427	0.068*	
C14A	−0.3807 (4)	−0.6389 (9)	−0.00639 (13)	0.0592 (14)	
H14A	−0.3146	−0.7240	−0.0020	0.071*	
C15A	−0.4803 (5)	−0.6641 (10)	0.00723 (13)	0.0683 (16)	
H15A	−0.4958	−0.7740	0.0230	0.082*	
O1C	−0.8218 (3)	0.0078 (6)	−0.44998 (13)	0.0703 (15)	
O2C	−0.7613 (2)	−0.4102 (6)	−0.38836 (8)	0.0505 (8)	
O3C	−0.9241 (2)	−0.1758 (5)	−0.39479 (7)	0.0472 (8)	
O4C	−0.9819 (2)	−0.2334 (6)	−0.31003 (7)	0.0522 (8)	
O5C	−1.0889 (3)	−0.5735 (6)	−0.32170 (8)	0.0488 (8)	
O6C	−1.1999 (3)	−0.4592 (7)	−0.25972 (11)	0.0637 (12)	
C1C	−0.7431 (5)	0.1642 (10)	−0.46269 (13)	0.0670 (15)	
H10	−0.7580	0.2719	−0.4788	0.080*	
C2C	−0.6432 (4)	0.1384 (9)	−0.44859 (13)	0.0594 (14)	
H11	−0.5771	0.2238	−0.4529	0.071*	
C3C	−0.6563 (4)	−0.0432 (10)	−0.42592 (15)	0.0525 (14)	
H12	−0.6004	−0.1010	−0.4125	0.063*	
C4C	−0.7649 (4)	−0.1166 (9)	−0.42743 (11)	0.0467 (12)	
C5C	−0.8333 (3)	−0.2969 (8)	−0.40988 (12)	0.0486 (12)	
H13	−0.8641	−0.4157	−0.4245	0.058*	
C6C	−0.8220 (4)	−0.5912 (10)	−0.37114 (13)	0.0545 (13)	
H14	−0.7716	−0.6637	−0.3559	0.065*	
H15	−0.8473	−0.7156	−0.3854	0.065*	
C7C	−0.9949 (3)	−0.3431 (8)	−0.37802 (11)	0.0492 (13)	
H16	−1.0294	−0.4539	−0.3927	0.059*	
H17	−1.0553	−0.2570	−0.3674	0.059*	
C8C	−0.9258 (4)	−0.4814 (8)	−0.35426 (16)	0.0446 (16)	
C9C	−0.8853 (3)	−0.3187 (9)	−0.32749 (11)	0.0486 (12)	
H18	−0.8349	−0.4075	−0.3138	0.058*	
H19	−0.8434	−0.1839	−0.3360	0.058*	
C10C	−0.9987 (4)	−0.6797 (8)	−0.33978 (13)	0.0540 (13)	
H10C	−1.0306	−0.7793	−0.3562	0.065*	
H10D	−0.9520	−0.7800	−0.3264	0.065*	
C11C	−1.0466 (4)	−0.4235 (10)	−0.29762 (12)	0.0445 (11)	
H11B	−1.0004	−0.5173	−0.2830	0.053*	
C12C	−1.1446 (4)	−0.3143 (9)	−0.28089 (11)	0.0502 (13)	
C13C	−1.1951 (4)	−0.0968 (11)	−0.28203 (12)	0.0587 (14)	
H13B	−1.1740	0.0319	−0.2946	0.070*	
C14C	−1.2866 (4)	−0.1037 (14)	−0.26034 (13)	0.0701 (15)	
H14C	−1.3370	0.0201	−0.2559	0.084*	
C15C	−1.2864 (4)	−0.3213 (13)	−0.24756 (13)	0.0708 (17)	
H15B	−1.3377	−0.3731	−0.2324	0.085*	

### Atomic displacement parameters (Å^2^)

**Table:**

	*U*^11^	*U*^22^	*U*^33^	*U*^12^	*U*^13^	*U*^23^
O1A	0.056 (2)	0.081 (3)	0.055 (3)	−0.0011 (19)	−0.010 (2)	0.012 (2)
O2A	0.0440 (17)	0.049 (2)	0.0598 (19)	0.0066 (15)	−0.0060 (16)	−0.0094 (16)
O3A	0.0468 (18)	0.047 (2)	0.055 (2)	0.0073 (16)	−0.0066 (17)	−0.002 (2)
O4A	0.0347 (16)	0.053 (2)	0.063 (2)	−0.0062 (14)	−0.0055 (16)	0.007 (2)
O5A	0.0325 (16)	0.052 (2)	0.0610 (18)	−0.0038 (13)	−0.0051 (15)	0.0122 (16)
O6A	0.045 (2)	0.091 (4)	0.070 (3)	0.0075 (18)	0.005 (2)	0.032 (2)
C1A	0.050 (3)	0.104 (6)	0.056 (4)	−0.002 (3)	−0.012 (3)	−0.006 (3)
C2A	0.059 (4)	0.077 (5)	0.083 (4)	−0.010 (3)	−0.010 (3)	−0.020 (4)
C3A	0.058 (3)	0.062 (4)	0.062 (3)	−0.007 (3)	−0.011 (3)	−0.004 (3)
C4A	0.043 (3)	0.058 (4)	0.047 (3)	0.002 (2)	−0.005 (2)	0.002 (2)
C5A	0.043 (3)	0.051 (3)	0.053 (3)	0.004 (2)	0.003 (2)	0.006 (3)
C6A	0.038 (3)	0.059 (3)	0.052 (3)	0.003 (2)	0.002 (2)	0.004 (2)
C7A	0.053 (3)	0.046 (3)	0.060 (3)	−0.003 (2)	−0.015 (3)	−0.002 (3)
C8A	0.032 (2)	0.040 (4)	0.050 (4)	0.0023 (18)	−0.004 (2)	−0.001 (2)
C9A	0.053 (3)	0.043 (3)	0.073 (4)	−0.006 (2)	−0.015 (3)	0.000 (3)
C10A	0.034 (2)	0.053 (3)	0.057 (3)	0.009 (2)	−0.007 (2)	0.005 (2)
C11A	0.042 (3)	0.052 (3)	0.046 (3)	0.000 (2)	−0.005 (2)	−0.001 (2)
C12A	0.036 (2)	0.053 (3)	0.040 (3)	−0.002 (2)	0.001 (2)	0.000 (2)
C13A	0.045 (3)	0.062 (4)	0.063 (4)	0.004 (2)	0.010 (3)	0.010 (3)
C14A	0.052 (3)	0.065 (4)	0.061 (4)	0.008 (2)	−0.014 (3)	0.002 (3)
C15A	0.058 (3)	0.081 (4)	0.066 (4)	−0.002 (3)	−0.007 (3)	0.025 (3)
O1C	0.0427 (19)	0.092 (4)	0.076 (3)	0.0014 (17)	−0.001 (2)	0.032 (2)
O2C	0.0375 (16)	0.052 (2)	0.062 (2)	0.0069 (15)	0.0041 (17)	0.008 (2)
O3C	0.0334 (16)	0.053 (2)	0.0553 (18)	0.0017 (14)	0.0044 (14)	0.0040 (16)
O4C	0.0429 (18)	0.051 (2)	0.062 (2)	−0.0053 (15)	0.0059 (16)	−0.0054 (17)
O5C	0.0437 (18)	0.0462 (19)	0.057 (2)	−0.0067 (16)	0.0043 (17)	−0.001 (2)
O6C	0.061 (2)	0.074 (3)	0.056 (3)	−0.0053 (18)	0.009 (2)	0.007 (2)
C1C	0.058 (3)	0.076 (4)	0.068 (3)	−0.001 (3)	0.012 (3)	0.032 (3)
C2C	0.052 (3)	0.062 (4)	0.064 (4)	−0.011 (2)	−0.001 (3)	0.004 (3)
C3C	0.041 (3)	0.065 (4)	0.051 (4)	−0.005 (2)	0.000 (3)	0.003 (3)
C4C	0.040 (3)	0.054 (3)	0.046 (3)	−0.001 (2)	0.001 (2)	0.002 (3)
C5C	0.037 (2)	0.051 (3)	0.058 (3)	0.003 (2)	0.004 (2)	−0.004 (2)
C6C	0.048 (3)	0.050 (3)	0.066 (3)	0.006 (2)	0.012 (3)	0.008 (3)
C7C	0.035 (2)	0.053 (3)	0.060 (3)	−0.003 (2)	−0.002 (2)	0.002 (2)
C8C	0.042 (3)	0.034 (3)	0.058 (4)	−0.0001 (18)	0.001 (3)	0.001 (2)
C9C	0.031 (2)	0.058 (3)	0.057 (3)	−0.002 (2)	0.004 (2)	−0.005 (2)
C10C	0.057 (3)	0.042 (3)	0.063 (3)	−0.004 (2)	0.012 (3)	0.000 (3)
C11C	0.044 (3)	0.048 (3)	0.042 (3)	0.002 (2)	−0.002 (2)	0.006 (3)
C12C	0.047 (3)	0.067 (4)	0.036 (3)	−0.005 (2)	−0.003 (2)	0.005 (2)
C13C	0.058 (3)	0.064 (4)	0.055 (3)	0.013 (3)	0.007 (3)	0.005 (3)
C14C	0.055 (3)	0.095 (5)	0.060 (4)	0.014 (3)	0.005 (3)	−0.011 (4)
C15C	0.048 (3)	0.106 (5)	0.059 (3)	−0.006 (3)	0.015 (3)	−0.011 (4)

### Geometric parameters (Å, °)

**Table:**

O1A—C4A	1.370 (6)	O1C—C4C	1.363 (6)
O1A—C1A	1.374 (7)	O1C—C1C	1.383 (6)
O2A—C5A	1.401 (6)	O2C—C5C	1.401 (5)
O2A—C6A	1.448 (5)	O2C—C6C	1.439 (6)
O3A—C5A	1.416 (6)	O3C—C5C	1.418 (5)
O3A—C7A	1.445 (5)	O3C—C7C	1.442 (5)
O4A—C11A	1.405 (5)	O4C—C11C	1.410 (6)
O4A—C9A	1.448 (6)	O4C—C9C	1.439 (5)
O5A—C11A	1.412 (5)	O5C—C11C	1.417 (6)
O5A—C10A	1.430 (5)	O5C—C10C	1.440 (6)
O6A—C15A	1.363 (6)	O6C—C15C	1.377 (7)
O6A—C12A	1.375 (6)	O6C—C12C	1.377 (6)
C1A—C2A	1.327 (9)	C1C—C2C	1.327 (7)
C1A—H1	0.9300	C1C—H10	0.9300
C2A—C3A	1.431 (7)	C2C—C3C	1.411 (8)
C2A—H2	0.9300	C2C—H11	0.9300
C3A—C4A	1.324 (7)	C3C—C4C	1.343 (6)
C3A—H3A	0.9300	C3C—H12	0.9300
C4A—C5A	1.486 (6)	C4C—C5C	1.491 (6)
C5A—H3	0.9800	C5C—H13	0.9800
C6A—C8A	1.512 (8)	C6C—C8C	1.545 (7)
C6A—H4	0.9700	C6C—H14	0.9700
C6A—H5	0.9700	C6C—H15	0.9700
C7A—C8A	1.526 (7)	C7C—C8C	1.512 (7)
C7A—H6	0.9700	C7C—H16	0.9700
C7A—H7	0.9700	C7C—H17	0.9700
C8A—C9A	1.528 (7)	C8C—C10C	1.531 (7)
C8A—C10A	1.532 (7)	C8C—C9C	1.536 (8)
C9A—H8	0.9700	C9C—H18	0.9700
C9A—H9	0.9700	C9C—H19	0.9700
C10A—H10A	0.9700	C10C—H10C	0.9700
C10A—H10B	0.9700	C10C—H10D	0.9700
C11A—C12A	1.484 (6)	C11C—C12C	1.487 (7)
C11A—H11A	0.9800	C11C—H11B	0.9800
C12A—C13A	1.328 (6)	C12C—C13C	1.352 (7)
C13A—C14A	1.427 (8)	C13C—C14C	1.420 (7)
C13A—H13A	0.9300	C13C—H13B	0.9300
C14A—C15A	1.315 (6)	C14C—C15C	1.332 (8)
C14A—H14A	0.9300	C14C—H14C	0.9300
C15A—H15A	0.9300	C15C—H15B	0.9300
			
C4A—O1A—C1A	105.7 (5)	C4C—O1C—C1C	105.7 (4)
C5A—O2A—C6A	111.5 (4)	C5C—O2C—C6C	110.7 (3)
C5A—O3A—C7A	110.7 (3)	C5C—O3C—C7C	110.6 (3)
C11A—O4A—C9A	110.7 (3)	C11C—O4C—C9C	111.8 (4)
C11A—O5A—C10A	110.5 (3)	C11C—O5C—C10C	112.0 (3)
C15A—O6A—C12A	106.0 (4)	C15C—O6C—C12C	105.5 (5)
C2A—C1A—O1A	110.7 (5)	C2C—C1C—O1C	110.2 (5)
C2A—C1A—H1	124.7	C2C—C1C—H10	124.9
O1A—C1A—H1	124.7	O1C—C1C—H10	124.9
C1A—C2A—C3A	106.2 (6)	C1C—C2C—C3C	107.0 (4)
C1A—C2A—H2	126.9	C1C—C2C—H11	126.5
C3A—C2A—H2	126.9	C3C—C2C—H11	126.5
C4A—C3A—C2A	106.9 (6)	C4C—C3C—C2C	106.9 (5)
C4A—C3A—H3A	126.5	C4C—C3C—H12	126.6
C2A—C3A—H3A	126.5	C2C—C3C—H12	126.6
C3A—C4A—O1A	110.5 (4)	C3C—C4C—O1C	110.2 (5)
C3A—C4A—C5A	133.5 (5)	C3C—C4C—C5C	134.0 (5)
O1A—C4A—C5A	116.0 (5)	O1C—C4C—C5C	115.8 (4)
O2A—C5A—O3A	112.1 (4)	O2C—C5C—O3C	111.8 (4)
O2A—C5A—C4A	107.0 (4)	O2C—C5C—C4C	108.0 (4)
O3A—C5A—C4A	108.0 (3)	O3C—C5C—C4C	108.2 (4)
O2A—C5A—H3	109.9	O2C—C5C—H13	109.6
O3A—C5A—H3	109.9	O3C—C5C—H13	109.6
C4A—C5A—H3	109.9	C4C—C5C—H13	109.6
O2A—C6A—C8A	111.3 (3)	O2C—C6C—C8C	110.6 (4)
O2A—C6A—H4	109.4	O2C—C6C—H14	109.5
C8A—C6A—H4	109.4	C8C—C6C—H14	109.5
O2A—C6A—H5	109.4	O2C—C6C—H15	109.5
C8A—C6A—H5	109.4	C8C—C6C—H15	109.5
H4—C6A—H5	108.0	H14—C6C—H15	108.1
O3A—C7A—C8A	110.3 (4)	O3C—C7C—C8C	110.8 (3)
O3A—C7A—H6	109.6	O3C—C7C—H16	109.5
C8A—C7A—H6	109.6	C8C—C7C—H16	109.5
O3A—C7A—H7	109.6	O3C—C7C—H17	109.5
C8A—C7A—H7	109.6	C8C—C7C—H17	109.5
H6—C7A—H7	108.1	H16—C7C—H17	108.1
C6A—C8A—C9A	110.8 (4)	C7C—C8C—C10C	109.9 (4)
C6A—C8A—C7A	107.1 (5)	C7C—C8C—C9C	111.4 (4)
C9A—C8A—C7A	110.0 (4)	C10C—C8C—C9C	107.5 (5)
C6A—C8A—C10A	110.9 (4)	C7C—C8C—C6C	108.3 (5)
C9A—C8A—C10A	107.7 (5)	C10C—C8C—C6C	110.1 (4)
C7A—C8A—C10A	110.2 (4)	C9C—C8C—C6C	109.7 (4)
O4A—C9A—C8A	110.2 (4)	O4C—C9C—C8C	109.7 (3)
O4A—C9A—H8	109.6	O4C—C9C—H18	109.7
C8A—C9A—H8	109.6	C8C—C9C—H18	109.7
O4A—C9A—H9	109.6	O4C—C9C—H19	109.7
C8A—C9A—H9	109.6	C8C—C9C—H19	109.7
H8—C9A—H9	108.1	H18—C9C—H19	108.2
O5A—C10A—C8A	110.4 (3)	O5C—C10C—C8C	109.3 (4)
O5A—C10A—H10A	109.6	O5C—C10C—H10C	109.8
C8A—C10A—H10A	109.6	C8C—C10C—H10C	109.8
O5A—C10A—H10B	109.6	O5C—C10C—H10D	109.8
C8A—C10A—H10B	109.6	C8C—C10C—H10D	109.8
H10A—C10A—H10B	108.1	H10C—C10C—H10D	108.3
O4A—C11A—O5A	110.9 (4)	O4C—C11C—O5C	111.2 (4)
O4A—C11A—C12A	107.2 (3)	O4C—C11C—C12C	106.9 (4)
O5A—C11A—C12A	108.7 (4)	O5C—C11C—C12C	108.7 (4)
O4A—C11A—H11A	110.0	O4C—C11C—H11B	110.0
O5A—C11A—H11A	110.0	O5C—C11C—H11B	110.0
C12A—C11A—H11A	110.0	C12C—C11C—H11B	110.0
C13A—C12A—O6A	109.6 (5)	C13C—C12C—O6C	110.1 (5)
C13A—C12A—C11A	134.8 (5)	C13C—C12C—C11C	133.7 (5)
O6A—C12A—C11A	115.6 (4)	O6C—C12C—C11C	116.2 (5)
C12A—C13A—C14A	107.0 (5)	C12C—C13C—C14C	106.5 (6)
C12A—C13A—H13A	126.5	C12C—C13C—H13B	126.7
C14A—C13A—H13A	126.5	C14C—C13C—H13B	126.7
C15A—C14A—C13A	106.4 (5)	C15C—C14C—C13C	106.9 (6)
C15A—C14A—H14A	126.8	C15C—C14C—H14C	126.6
C13A—C14A—H14A	126.8	C13C—C14C—H14C	126.6
C14A—C15A—O6A	111.0 (5)	C14C—C15C—O6C	110.9 (5)
C14A—C15A—H15A	124.5	C14C—C15C—H15B	124.5
O6A—C15A—H15A	124.5	O6C—C15C—H15B	124.5

### Hydrogen-bond geometry (Å, °)

**Table:**

*D*—H···*A*	*D*—H	H···*A*	*D*···*A*	*D*—H···*A*
C1A—H1···Cg1	0.93	2.70	3.478 (6)	142
